# The Genus *Ferulago*: A Review on Ethnopharmacology, Phytochemistry, and Pharmacology

**DOI:** 10.22037/ijpr.2021.114754.15020

**Published:** 2021

**Authors:** Yahya Rahimpour, Abbas Delazar, Solmaz Asnaashari, Parina Asgharian

**Affiliations:** a *Student Research Committee, Tabriz University of Medical Sciences, Tabriz, Iran.*; b *Immunology Research Center, Tabriz University of Medical Sciences, Tabriz, Iran. *; c *Drug Applied Research Center, Tabriz University of Medical Sciences, Tabriz, Iran. *; d *Biotechnology Research Center, Tabriz University of Medical Sciences, Tabriz, Iran. *; e *Department of Pharmacognosy, School of Pharmacy, Tabriz University of Medical Sciences, Tabriz, Iran.*

**Keywords:** Ferulago, Essential oil, Pharmacological activity, Herbal medicine, Phytotherapy

## Abstract

The Ferulago genus appertains to the Umbelliferae family comprises 49 species which are mainly distributed in Asia, Europe, and Africa. This paper aims to review the morphological properties of *Ferulago* species, herbal components, and their pharmacological properties.

The information of this review paper has been collected from journals available in databases including Science Direct, Web of Science, Scopus, PubMed, EBSCO, Google Scholar, and Hindawi up to March 2020. In traditional medicine, the genus of *Ferulago* has been used to treat intestinal worms, snake bites, wound skin infections, diseases of the spleen and gastrointestinal tract, and headaches. It not only has been used traditionally as a preservative agent to dairy, oil ghee, and meat but also has given them a pleasant taste. The main components of *Ferulago* spp. are monoterpenes, sesquiterpenes, coumarin, furanocoumarin, flavonoids, and terpenoids have been the reason for the antimicrobial, antioxidant, anticoagulant, antidiabetic, Alzheimer, and larvicidal properties of this plant. This review confirms that many traditional uses of some *Ferulago* species have now been validated by modern pharmacology research. Rigorous investigations of all the species of *Ferulago* concerning phytochemical and pharmacological properties, mainly their mechanism of action, efficacy, and safety might offer a context for researchers to prosper plant-derived medications like anti-diabetes, antibiotics, and sedatives treating drugs, and the key to directing clinical trials.

## Introduction

Herbal used in traditional and modern medicine presents a valuable source of secondary metabolites and different pharmacological and biological activities. Hence, they could be utilized as a lead compound to produce new drugs and treat numerous diseases (1). The Apiaceae or Umbelliferae, commonly known as the parsley, carrot, or celery family, is one of the biggest plant families in the world usually characterized by aromatic plants with hollow stems (2). Several plants belonging to this family are well-known as vegetables, culinary, and medicinal plants, including *Apium gravolence*, *Foeniculum vulgare*, *Centella asiatica*, *Pimpinella anisum*, *Cuminum cyminum*, *Carum carvi*, *Ligusticum officinale*, *Coriandrum sativum*, *Anethum graveolens* , *Ammi visnaga*, *Anthriscus cerefolium*, *Ferulago angulata*, and *Ferula assa-foetida* (3). Plants of the Apiaceae family are commonly aromatic and pungent owing to the existence of essential oil or oleoresin in their diverse organs (4). Among several Umbelliferae families, the species belonging to the genus *Ferulago* W. Koch. are broadly employed in traditional medicine. The genus *Ferulago* W. Koch. (called Chavil or Chavir in Persian, Çakşır or Çağşır in Turkey) appertains to the Umbelliferae family consisting of 49 species which are mostly distributed in Asia, Europe, and Africa (5, 6).The occurrence of several bioactive secondary metabolites, including coumarin, coumarin esters, furanocoumarin, flavonoids, quinones, steroids, essential oils, steroids, and terpenoids are owing to their pharmacological activities (7). Extensive studies have been conducted to evaluate the ethnomedicinal uses of *Ferulago* species antibacterial, antioxidant, Alzheimer, anti-diabetics, anti-malaria, anti-coagulant, and aphrodisiac effects. The information of this review paper has been collected from journals available in databases including Science Direct, Web of Science, Scopus, PubMed, EBSCO, Google Scholar, and Hindawi up to March 2020. The keywords and search terms contained “*Ferulago*”, “*Ferulago* spp.” “essential oil of *Ferulago*”, “phytochemistry and Ferulago”, “coumarins and *Ferulago”, “*bioactive compounds and* Ferulago”, “*pharmacological activity and* Ferulago”. *Therefore, we conducted the present review article to provide a complete overview of a current state of knowledge on botany, ethnomedicinal uses, phytochemistry, and the most noticeable pharmacological effects of species belonging to the genus *Ferulago*. 

**The botany of Ferulago plants**

**Figure 1:**
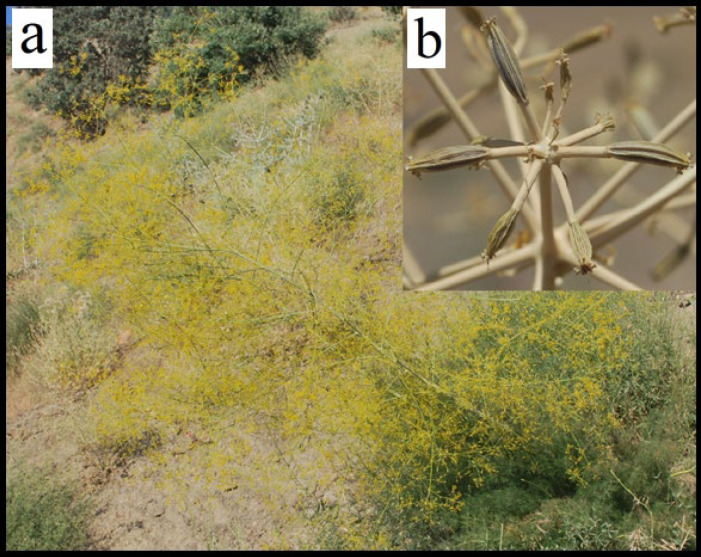
(a) The *Ferulago bernardii* in the flowering stage, (b) the fruits of the *Ferulago stellata*

The Ferulago genus appertains to the Umbelliferae family and comprises 49 species which are mainly distributed in Asia, Europe, and Africa (8). The genus *Ferulago* is distributed in Turkey with 34 species, out of which 18 are endemic (9). The 8 species exist in Iran, out of which three are endemic (10). According to The Plant List website, the genus *Ferulago* consists of 48 accepted species names and 13 unassessed species(11). Table 1 depicts the name of accepted species and their synonyms. *Ferulago* genus is 60-150 cm tall; annual or perennial plants grow at altitudes of 1900- 3200m above sea level with yellow fruits. They are characterized by persistent bracts and bracteoles and small flowers which are widely distributed in Turkey, Iraq, and west of Iran (from the flora of Iran, particularly in Kermanshah, Ilam, Lorestan, and Kurdistan) (8, 12, 13). Pictures of *Ferulago bernardii *and fruits of* ferulago stellata* are shown in Fig.1. The high distribution of *Ferulago* spp. in these areas suggests that the core center of the biodiversity of the genus Ferulago is in Anatolia. Based on taxonomy the species of this genus resemble *Prangos* and *Ferula* species. They are widely used in the Anatolia region for several purposes (7, 14). The most studied species is *F. angulata* which is divided based on ovaries, the flowering of in fluorescence, and fibers (rather absence or presence of trichomes) into two subspecies: *F. angulate* subsp. *angulata* and* F. angulate* subsp. *Carduchorum* by Chamberlain in 1987(15).

**Traditional use of** ***Ferulago*** **spp.**

*Ferulago* like *Ferula* and *Prangos* species have been traditionally utilized back in history in folk medicine for the medicament of hemorrhoids, and intestinal worms, peptic, sedative, carminative, digestive, aphrodisiac, snakebite, wound skin infections, headache, diseases of the gastrointestinal tract and spleen (5, 16). Due to the aromatic property of *Ferulago*, some of them have been applied in Iranian tradition as spices in different foods, such as dairy and meat products, for increasing the flavor and as an aroma. They have also been employed as natural preservatives for enhancing food expiration dates (8, 16 and 17). Bakhtiari nomads use *F. angulata* to make some foods and Nomads of Fars province utilize this plant to flavor yogurt. The *F. angulata* is also used as a sedative, food-digestive, tonic, antibacterial, and antiparasitic(18). In Turkish traditional medicine, the aerial parts of a few members of this genus are used as an immunostimulant, tonic, sedative, digestive, anti-bronchitis, flavor, vermicidal, anthelmintic, and anti-peptic (19, 20). Likewise, the root parts are used for the treatment of dermatological disorders and cancers and as an aphrodisiac and are preferred as fodder to improve animal productivity (7). The seeds are applied for eye pains in the form of inhalation (8). It has been reported that dried or fresh leaves of *Ferulago* are used as foot deodorant by indigenous people of the north of Iraq (21).

**Phytochemistry of essential oils from the genus Ferulago species**

Essential oils or volatile oils are the phytochemical complexes of different aromatic components, mainly monoterpenoids and sesquiterpenoids, which are obtained from plant materials, for instance, leaves, fruits, seeds buds, flowers, roots, and bark. They are characterized by having a strong odor generally lower density than water, being volatile, rarely colored, liquid, lipophilic, and soluble in organic solvents (7). Despite the way that these volatile oils involve around 20–60 constituents, just a few of them exist at high amounts (20–70%) in correlation with different constituents existing in low sums (22). These phytochemicals assume a vital part in the protection of the herbs from herbivores, insects, bacteria, fungi, viruses, and also help to attract pollinators (23). These essential oils could be extracted with conventional methods (steam distillation, hydrodistillation (HD), organic solvent extraction) and innovative techniques (In situ microwave-generated hydrodistillation, supercritical carbon dioxide, microwave steam diffusion, microwave hydro diffusion and gravity, and microwave steam distillation). Among these methods, the hydro-distillation method is the most prevalent technique for obtaining essential oils (7, 24 and 25). The method of extraction, drying methods, genotypic variation, geographical origin of the plant, stage of the development, and part of the plant used may drastically affect the composition of essential oils of plants (26-28). Based on the literature, researches have revealed that the species of the *Ferulago* genus are appropriate for the extraction of essential oils and many of them have been evaluated for chemical compositions, including *F. macedonica*, *F. angulata, F. carduchorum*, *F. phialocarpa*, *F. contracta*, *F. macrocarpa*, *F. blancheana*, *F. bernardii*, *F. pachyloba*, *F. longistylis*, *F. isaurica*, *F. syriaca*, *F. platycarpa*, *F. thyrsiflora*, *F. sylvatica*, *F. nodosa*, *F. pauciradiata*, *F. asparagifolia, F. aucheri*, *F. galbanifera, F. confusa, F. humilis, F. campestris, F. idaea, F. macrosciadia, F. mughlae, F. sandrasica, F. silaifolia, F. trachycarpa, F. thirkeana, F. setifolia, F. subvelutina, F. stellata, F. capillaries F. trifida.* Table 2 represents the main compounds of these essential oils, part of the herb that was used, methods of extraction, percentage yield, class of components, and their references. 

According to data of the volatile oils extracted from various *Ferulago* spp., it is clear that every species and each part of the plant have a diversified set of main compounds. Therefore, it is hard to find the similarity among the species of this genus concerning the chemicals. Several compounds, like α-Pinene, p-Cymene 2,3,6-trimethyl benzaldehyde, cis-Chrysanthenyl acetate, α-Phellandrene, Sabinene, (Z)-β-Ocimene, Limonene, Myrcene, Terpinolene, Nonacosane, and δ-Cadinene, have been detected as main components of the volatile oils of many *Ferulago* species. To date, analysis has shown that the α-Pinene is a major compound of several *Ferulago* species; hence, this might be regarded as a perpetual constituent for the genus (7). There are certain other notable points to report; primarily, the compound of spathulenol was the major component from aerial parts of only two species namely, *F. sylvatica* and *F. thyrsiflora* (45). Secondly, the compound of ferulagone was found as the main compound of only *F. thirkeana* obtained from its fruits (59). Thirdly, carvacrol methyl ether obtained with MD from fruits of *F. macrosciadia* and *F. idaea* was the major component of only these two species (47). Furthermore, Cecchini et al. compared the composition of the volatile oil from roots and fruits of *F. campestris* from two sites of collection and two periods of time. They found significant differences in the percentages of (2, 30 and 6) ‐trimethyl benzaldehyde (14.8–27.9% in the roots gathered in the summer, 65.2% in roots gathered in the fall) and α-Pinene (58.3-75% in the roots gathered in the summer, 19.3% in the roots gathered collected in the fall) (23). 

**Phytochemistry of plant extracts from the genus Ferulago species**

Phytochemical investigations on *Ferulago* species have shown the presence of various secondary metabolites including coumarins, coumarin esters, furanocoumarins, aromatic compounds, monoterpenes, sesquiterpenes, flavonoids, quinones, and stilbene. Table 3 depicts the main phytochemicals that have been isolated and characterized from *Ferulago* species. Up to now, several studies have been done to distinguish active compounds from different parts of the *ferulago* genus, from which about 73 (three simple coumarins, sixteen furanocoumarins, five dihydro-furanocoumarin, four sesquiterpene coumarin, twelve prenylated coumarins, six pyranocoumarin, nine flavonoids, and eighteen miscellaneous compounds) bioactive compounds were isolated. Based on the literature, coumarins and their derivatives are the most prevalent secondary metabolites on the *Ferulago* species and might be used as a chemotaxonomic marker in the genus *Ferulago* (65). The classification of different types of coumarins and various biological applications of each compound was reviewed by Venugopala *et. al.* (66). 

**Pharmacological activities**

In the last two decades, many *ferulago* species have been widely studied with advanced scientific methods and reported for several pharmacological properties such as antibacterial activity, antioxidant activity, antidiabetic activity, larvicidal activity, Alzheimer, and anticancer activity. These pharmacological activities of Ferulago are considered to be attributed mainly to its coumarins and furanocoumarins and essential oil (66). Table 4 depicts pharmacological activity and model of study of the genus *Ferulago*. 

**Antibacterial activity**

The volatile oils of many herbs of genus *Ferulago* have been the focus on pharmacological activity, particularly from an anti-oxidant, antimicrobial and antifungal point of view (7, 20 and 29). In the literature, antimicrobial and antifungal properties of essential oil were screened versus Gram-positive (*Staphylococcus aureus*, *S. epidermidis, and methicillin-resistant S. aureus*), and Gram-negative (*Escherichia coli, Salmonella typhimurium, Bacillus cereus, Proteus vulgaris, Enterobacter aerogenes, and Pseudomonas aeruginosa*) bacteria, and the yeast (*Candida albicans, C. parapsilosis, *and* C. tropicalis*) via broth microdilution assay. Sucu et al., 2019, studied the antimicrobial effects of volatile oil from the roots and aerial parts of *F. sandrasica* and found that both essential oils were not active against *C. tropicalis* and *C. parapsilosis* compared to positive controls (55). The volatile oil of the aerial portions was found to be active against *Salmonella typhimurium, Staphylococcus aureus*, and *Bacillus subtilis*, however, inactive against *E. coli;* the root essential oil was active against *B. subtilis* and *S. typhimurium* compared with *E. coli*, but not active against *S. aureus*. Recently, Karakaya et al. assessed antimicrobial activity of n-butanol, ethyl acetate, dichloromethane, methanol extracts, and aqueous residue parts of methanol extracts from the aerial parts and roots of four *Ferulago* species (*F. pachyloba, F. bracteata, F. trachycarpa, *and* F. blancheana*) along with 14 isolated compounds via micro broth-dilution methods. Their result demonstrated that the best antimicrobial effect against *B. subtilis, E. coli, S. aureus, P. aeruginosa, *and* C. albicans* were obtained with methanol extract of the roots, n-butanol fractions, and methanol extract of the aerial parts from *F. blancheana* (62.5 µg/mL), dichloromethane fraction of the roots and aerial parts from *F. pachyloba* (62.5, 31.25 µg/mL), the n-butanol fraction of the aerial parts, dichloromethane fractions of the aerial parts and roots, methanol extracts and ethyl acetate fraction of the roots from *F. bracteata* (62.5 µg/mL), dichloromethane fraction of roots, and methanol extracts of roots and aerial parts from *F. trachycarpa* (62.5 µg/mL) and prantschimgin (31.25 µg/mL) (78). According to them, the *E. coli* was less affected than the other microorganisms; the best activity against *C. albicans* (MIC = 31.25 μg/mL) was obtained by the CH_2_Cl_2_ fraction of aerial portions from *F. pachyloba* and isolated compound prantschimgin. Furthermore, Pinto et al. evaluated the antifungal effects of an essential oil, and two main compounds of it on germ tube formation, ergosterol biosynthesis, and mitochondrial function. Limonene presented a weaker activity (0.32 to 20 μL/mL) than the essential oil and α-pinene with low and similar to MIC and MFC values against the tested organisms (0.08 to 5.0 μL/mL). The essential oil of *F. capillaris* suppressed germ tube formation at sub-inhibitory dose on *Candida albicans*. The mechanism of antifungal activity of *F. capillaris* indicated no distribution on the ergosterol content and defect of mitochondrial role in a dose-dependent way in essential oil-treated *C. albicans* (62).

**Anti-oxidant effect**

In recent decades, extensive studies have been conducted on the evaluation of the antioxidant function of medicinal plants as a source of natural compounds not only to combat several degenerative disorders, including cardiovascular disease and cancer, but also as a substitute compound to artificial additives, like butylated hydroxytoluene (BHT) and butylated hydroxyanisole (BHA) in food productions (94). Anti-oxidant constituents diminish the extent of oxidative damage via acting as free radical scavengers (95). There are numerous methods, such as radical scavenging power, reducing power, and inhibition of lipid peroxidation in a β-carotene–linoleate system for assessing the antioxidant activity of plant extracts or volatile oils (96). The DPPH radical-scavenging method is one of the easiest and rapid tests for evaluating the antioxidant activity of natural compounds(16). According to literature, an antioxidant activity study has been performed on *F.macrocarpa, F. carduchorum* (97), *F. bernardii* (16), *F. sandrasica, F.macedonica, F. trifida* (64), *F. subvelutina* (74), *F. cassia* (8), *F. angulata*, and *F. campestris* (79). The antioxidant activities of four fractions and crude extract of aerial portions of *F. carduchorum* at 2 vegetative periods (flower and fruit) were assessed using the DPPH method. The best activity belonged to flower crude extract (IC_50 _= 0.44 mg/mL)(97). Shahbazi and Shavisi investigated the antioxidant activities of nonpolar and polar sub-fractions of methanolic extract, and the volatile oil of the aerial parts of *F. bernardii* and compared them to BHT via DPPH assay. The antioxidant activity with the mean of IC_50 _were polar sub-fractions (5.66), non-polar sub-fractions (6.88), and essential oil (14.81), while they displayed lower radical scavenging activity compared with BHT(16). Tavakoli *et al.* found that due to lack of phenolic compounds in the conformation of the volatile oil of different parts of *F. trifida*, a feeble free radical scavenging effect was observed (IC_50_: 95–120 μg mL^−1^) compare to BHT (IC_50_: 21.2 ± 2.6 μg mL^−1^) (64). Moderate antioxidant activities were attained with DPPH test from isolated coumarin of the roots of *F. subvelutina* compare to BHT (IC_50 _= 27 µg/mL) > oxypeucedanin hydrate (IC_50_= 160 µg/mL) >meranzin hydrate (IC_50 _= 180 µg/mL) >osthole(IC_50 _= 27 µg/mL) >oxypeucedanin (IC_50_= 217 µg/mL) >isoimperaturin(IC_50 _= 245 µg/mL) > xanthotoxin (IC_50_= 270 µg/mL) (74). In another study, the antioxidant activity of isolated compound, and the extracts and fractions of the aerial parts, fruits, flower, and roots of *F. cassia* were investigated via TBA assay. The highest antioxidant potential was obtained based on following order: Peucedanol (IC_50 _= 18.1 µg/mL)>Suberosin (IC_50 _= 23.5 µg/mL)> roots CH_2_Cl_2_fractions (IC_50 _= 43.1 µg/mL)> fruit CH_2_Cl_2_ fractions (IC_50 _= 54.4 µg/mL) > Grandivitinol (IC_50 _= 61.1 µg/mL) > Umbelliferone (IC_50 _= 79.5 µg/mL) (8).

**Alzheimer’s disease (AD)**

Alzheimer’s is one of the neurological disorders with decreasing acetylcholine followed by the deterioration of short-term memory, which occurs in elderly people mostly based on genetic inheritance. One promising strategy for combating AD is using anticholinesterases or acetylcholinesterase inhibitors (AChEIs) to inhibit the hydrolysis of acetylcholine and raise its level in the synaptic cleft (82). Hajimehdipoor and co-worker studied the acetylcholinesterase inhibitory property of the total extracts and fractions of the aerial portions of *F. subvelutina *and the whole plant of *F. angulata* employing the Ellman method. Their result implied that a total extract of both *Ferulago* genes can inhibit the acetylcholinesterase enzyme with 19.7% and 15.8% respectively for *F. subvelutina* and *F. angulata.* The dichloromethane fraction displayed the highest AChEI activity among all the fractions (98). Golfakhrabadi *et al.* found that all the coumarins obtained from the aerial parts of *F. carduchorum* have AchE enzyme inhibition, among which xanthotoxin revealed the highest inhibitory (IC_50 _= 39.64 µM) (70). The essential oil of *F. carduchorum *has been also found to be of AChE inhibitory activity (IC_50 _= 23.6 µL mL^-1^) (34). In another study on AChE inhibitors via bioassay-guided isolation, the dichloromethane extract from the root of *F. campestris* was investigated. Three daucane ester derivatives (1-acetyl-5-angeloyl lapiferol, ferutinin, and siol anisate), two phenol derivatives (epielmanticine and 2-epilaserine), one polyacetylene (9-epoxyfalcarindiol), and three coumarin derivatives (coladin, coladonin, umbelliprenin) were isolated. All the obtained constituents could inhibit the AChE, but at higher doses (IC_50 _1.2–0.1 mM) than the standard galantamine (6.7 μM) and the most active compounds were the epielmanticine and the siol anisate with IC_50_ of 0.172 and 0.175 µM, respectively (82). In addition, Karakaya and co-workers studied the anticholinesterase effects of the fractions and extracts from the aerial parts and roots of *F. isaurica* and *F. syriaca*. Strong inhibitory activities against butyrylcholinesterase (BuChE) (88.56 ± 2.34%) and AChE (46.99%) at 20 µg/mL were observed in the CHCl_3_ part of the root of *F. Isaurica*. Felamidin (77.11%); prantschimgin (74.82%), two obtained constituents from the chloroform part of roots, presented a strong inhibitory effect against BuChE (19). In another study, AChE inhibitory activities of the volatile oils of the fruits, roots, and flowers of *F. trifida* were investigated. The result revealed significant AChE inhibitory activity (78.7, 74.3, and 72.1% inhibition of Ach Enzyme, respectively) (64). Hritcu and co-worker found the volatile oil extracted from the aerial part of *F. angulata* has an anti-amnesic activity in scopolamine-induced memory deterioration in rats and diminish AChE activity in hippocampal (99). In another study the volatile oil from fruits of *F.pauciradiata* revealed strong inhibitory properties against BuChE and AChE (IC_50_ = 0.567, 7.987 L/mL, respectively) (20). 

**Anti-diabetic effects**

α-glucosidase, and α-amylase inhibitory activity of the extracts and some compounds from the roots of *F. blancheana, F. trachycarpa, and F. pachyloba* have been evaluated by *in-vitro* bioassay-guided isolation methods to determine the anti-diabetic properties of this plant. The obtained result demonstrated that the highest activities versus α-glucosidase with an IC_50_ value of 0.3, 2, 2 mg/mL belonged to CH_2_Cl_2_ extracts of the roots of* F. trachycarpa*, *F. pachyloba, and F. blancheana,* respectively. Suberosin and felamidin compounds possessed momentous α-glucosidase inhibitory activities with IC_50_ values of 0.9 and 0.4 mg/mL, respectively, while the IC_50 _for acarbose as standard was 4.9 mg/mL. The acarbose depicted a strong α-amylase inhibitory activity (82.3%) at a dose of 1 mg/mL whereas none of the other extracts displayed a significant α-amylase inhibitory effect (100). 

**Anti-coagulant activity**

Golfakhrabadi *et al.* evaluated the toxicological profile of oral application of the *F. carduchorum* extract and the anticoagulant effects of two isolated coumarins (suberenol and suberosin) in male Wistar rats. The LD_50_ of the plant extract for acute toxicity was over 2000 mg/kg and there were no substantial variations (*p* > 0.05) among the control and the treated groups concerning the biochemical and hematological parameters. The prothrombin time (PT) of the treated group with a total extract from the aerial part of the plant has not shown a major impact relative to the control group (receiving tap water by gavage) (*p* > 0.05) at doses of 250 and 500 mg/kg. Meanwhile, suberosin expanded the PT at doses of 3 and 6 mg/kg (16.7 and 17.4 s, respectively) and suberenol at the same dose (16.5 and 17.1 s, respectively) (17).

**Anti-malaria**

Khanavi *et al*. assessed the larvicidal activity of chloroform, methanol, ethyl acetate, and the total 80% methanol extract from the aerial part of *F. carduchorum* against late 3^rd^, and early 4^th^ instar larvae of the malaria vector Anopheles stephensi. The LC_90_ of chloroform, ethyl acetate, the total extract, and methanol fractions were 0.455, 1.892, 1.509, and 10.886 ppm, respectively. Moreover, the LC_50_ of chloroform, ethyl acetate, the total extract, and methanol fractions were 0.236, 0.744, 0.480, and 3.702 ppm, respectively. The chloroform fraction indicated lower LC_50_ and LC_90_ values than the other extracts, which might be due to the presence of high content of phytosteroids and coumarins (101). In another study, the larvicidal activity of a few isolated coumarin, methanol, and chloroform extracts of the roots, leaves, and fruits of *F. trifida* were investigated on the third instar larvae of A. stephensi Listonas. Strong insecticidal properties were found for methanol extract of the fruit with LC_90_ and LC_50_ values of 18.12 and 2.94 ppm, respectively. Among pure compounds, oxypeucedanin presented moderate toxicity against A. stephensi with LC_90_ and LC_50_ values of 346.41 and 116.54 ppm, respectively. It could be concluded that the methanol extract from the fruit of *F. trifida* might be utilized as an effective bio-insecticide in green control programs of mosquitoes, particularly *A. stephensi* (102).

**Aphrodisiac activity**

An in-vitro study was conducted by Ozturk et al. to investigate the aphrodisiac activity of the lyophilized water extract from the roots of *F. syriaca* on the human corpus cavernosum. For finding the mechanism involved in relaxation, the effect of the extract was investigated on the relaxing responses to selective guanilate cyclase inhibitor (oxadiazolo [4,3-α] quinoxalin-1one (ODQ)), NO-synthesis inhibitor (NG-nitro-L-arginine methyl ester (L-NAME)), forskolin, Electrical field stimulation (EFS), sodium nitroprusside, and acetylcholine. The result displayed that the extract could have to relax in a dose-dependent way on corpus cavernosum strips, and the L-NAME (small dosages) and QDQ (in all dosages) were able to suppress the extract-induced relaxation in the human corpus cavernosum. The *F. syriaca* extract enhanced the relaxation response of strips with Ach incubation while the extract did not affect the relaxation induced by EFS, forskolin sodium, and nitroprusside. The result suggested that the extract of *F. syriaca* could persumabely act via stimulating the NO- cyclic guanosine monophosphate (cGMP) pathway (103). 

**Toxicity profile of the genus** ***Ferulago***

Nowadays, the usage of herbal remedies has increased around the world, and patients falsely feel that they are healthy because they are natural. While these products comprise several bioactive compounds and might cause adverse effects on consumers (104, 105). Therefore, it is essential to herbal medicines undergo current safety and efficacy tests. Based on the literature review, there is only one piece of scientific study about the safety of this herb. Golfakhrabadi *et al.* investigated oral acute, and sub-chronic toxicities of the total extract of the aerial parts of *F. carduchorum *in the rat (17). To study acute toxicity five rats were treated with one dose of the total extract (2000 mg/kg) orally and the control group was treated with tap water. For the sub- chronic study, the doses (250, 500, and 1000 mg/kg) of the total extract were applied to treated groups by gavage for thirty following days. During the acute toxicity study, the animals did not show any signs of side effects, mortality, and the LD_50_ was over 2000 mg/kg in rats. The result of sub-chronic toxicity demonstrated that the total extract did not yield momentous changes in behavior, water, and food intake, breathing, body weight gain, blood metrics, and gastrointestinal properties in rats (17). This study indicated that *F. carduchorum* may be a healthy additive for conventional applications. 

**Table 1:** The accepted genus of Ferulago species based on The Plant List

No.	***Ferulago *** **species**	**Synonyms**
1	*F.* *angulata* (Schltdl.) Boiss.	F. angulata Schltdl., F. linearifolia Boiss., F. trifida Boiss.
2	F. angulata subsp. carduchorum (Boiss. & Hausskn.) D. F. Chamb	F. carduchorum Boiss. & Hausskn., F. abbreviata C. C. Towns.,
3	F. antiochia Saya & Miski	
4	*F.* *armena* (DC.) Bernardi	*F.* *pauciradiata* Boiss. & Heldr., *Ferula* *armena* DC., *F.* *bourgaei* Boiss. *Ferula* *gandogeri* M.Hiroe. *Ferula* *kochii* M.Hiroe. *F.* *sintenisii* Gand.
5	F. aucheri Boiss.	Ferula taurica (Schlschk.) M. Hiroe, F. galbanifera var. brachyloba Boiss., F. taurica Schischk.
6	F. asparagifolia Boiss.	F. longistylis Boiss.
7	F. bernardii Tomk. & Pimenov	
8	*F.* *biumbellata* Pomel	*Ferula* *sulcata* var. *biumbellata* (Pomel), *Ferula* *lutea* var. *mouretii* (Maire) Maire. Batt. *Ferula* *sulcata* var. *mouretii* Maire
9	*F. blancheana *Post ex Boiss.	
10	*F. bracteata *Boiss. & Hausskn	
11	*F. brachyloba *Boiss. & Reut.	*Ferula brachyloba *(Boiss. & Reut.) Nyman
12	*F. cassia *Boiss.	*F. amani *Post ex Boiss.
13	*F. contracta *Boiss. & Hausskn	
14	F. daghestanica Schischk.	
15	F. fieldiana Rech. f.	Ferula fieldiana (Rech.f.) M. Hiroe
16	*F. galbanifera *(Mill.) W. D. J. Koch	*Ferula daghestanica *(Schischk.) M. Hiroe, *Ferula campestris *Besser, *Ferula meoides *L., *Ferula galbaniifera *Mill, *F. campestris *(Besser) Grecescu, *F. meoides *(L.) Boiss. *F. dodonaei *Kostel,
17	*F.* *glareosa* Kandemir & Hedge	
18	*F. granatensis *Boiss.	*Ferula granatensis *(Boiss.) Steud.
19	*F. idaea *Özhatay & Akalin	
20	*F. humulis *Boiss.	*F. pumila *Boiss.
21	*F. isaurica *Peşmen	
22	*F.* *kurdica* Post	
23	*F.* *lutea* (Poir.) Grande	*Elaeoselinum* *mangenotianum* Emb., *Ferula* *capillaris* Link ex Spreng., *Ferula* *capillifolia* Link., *Ferula* *lutea* (Poir.) Maire, *Ferula* *lutea* var. *microcarpa* (Maire)Maire, *Ferula* *pomelii* M.Hiroe, *Ferula* *sulcata* Desf., *Ferula* *sulcata* var. *crassicosta* (Pomel) Batt., *Ferula* *sulcata* var. *leptocarpa* (Pomel) Batt., *Ferula* *sulcata* var. *microcarpa* Maire, *Ferula* *sulcata* var. *parvifolia* (Pomel) Batt. *F.* *barrelieri* Guss., *F.* *capillaris* (Link ex Spreng.) Cout., *F.* *capillifolia* (Link) Franco, *F.* *communis* Petter ex Nyman, *F.* *crassicosta* Pomel, *F.* *leptocarpa* Pomel *F.* *nodiflora* Koch, *F.* *parvifolia* Pomel, *F.* *sulcata* (Desf.) Ledeb., *Ligusticum* *luteum* Poir.
24	*F. macrocarpa *(Fenzl) Boiss.	*Ferula lophoptera *(Boiss.) Benth. & Hook. f., *Ferula mesopotamica*, *F. lophoptera *Boiss.
25	*F. macedonica *Micevski & E. Mayer	
26	*F.* *macrosciadea* Boiss. & Balansa	
27	*F. mughlae *Peşmen	
28	*F. nodosa *(L.) Boiss.	*Ferula cretica *(Spreng.) M. Hiroe, *Ferula geniculata *Guss., *Ferula nodosa *(L.) Benth. & Hook. f., *Ferula rigida *Ten., *Peucedanum nodosum *L,* F. geniculata *Boiss.
29	*F. pachyloba *(Fenzl) Boiss.	*Ferula cappadocica *(Bornm.) M. Hiroe, *Ferula pachyloba *Fenzl,, *F. cappadocica *Bornm.
30	*F. phialocarpa *Rech. f. & Riedl	
31	*F. sandrasica *Peşmen & Quezel	
32	*F. platycarpa *Boiss. & Balansa	*F. asperula *Freyn & Sint
33	*F.* *sartorii* Boiss.	
34	*F.* *scabra* Pomel	*Ferula* *algiersia* M.Hiroe, *Ferula* *sulcata* var. *scabra* (Pomel) Batt.
35	*F.* *serpentinica* Rech.f.	
36	*F. setifolia *K. Koch	
37	*F. stellata *Boiss.	*Ferula pauciflora *K. Koch, *Ferula setifolia *K. Koch, *Ferula sylvatica *Szov. ex Boiss., *F. oxyptera *Boiss., *F.* *pauciflora *K. Koch
38	*F. silaifolia *(Boiss.) Boiss.	Ferula silaifolia (Boiss) M. Hiroe
39	*F. subvelutina *Rech. f	*Ferula subvelutina *(Rech. f.) M. Hiroe, *Ferula turcomanica *(Schischk.)M. Hiroe, *F. turcomanica *Schischk
40	*F. sylvatica *(Besser) Rchb.	*Ferula barrelieri *Ten., *Ferula commutata *(Racher) M. Hiroe, *Ferula ferulago *var. *commutata *Rochel, *Ferula microphylla* M.Bieb. ex Schur, *Ferula* *monticola* (Boiss. & Heldr.) Neilr., *Ferula* *myriophylla* M.Bieb. ex Besser, *Ferula* *schischkinii* M.Hiroe, *Ferula* *sylvatica* Besser, *Ferula* *verticillata* Maerkl. *F.* *athamantifolia* Schur, *F.* *commutata* (Rochel) Degen, *F. latiloba* Schischk., *F. monticola* Boiss. & Heldr., *F.* *orphanidis* Boiss. & Heldr., *F.* *rocheliana* Nyman, *F. transsilvanica* Schur
41	*F.* *sylvatica* subsp. *confusa* (Velen.) Hartvig	*F. confuse* Velen.
42	*F. syriaca *Boiss	*F.* *cypria* H.Wolff
43	*F.* *ternatifolia* Solanas, M.B.Crespo & García-Martín	
44	*F. thirkeana *Boiss	
45	*F. trachycarpa *Boiss.	*Ferula insularis* (H. Wolff) M. Hiroe, *F. frigida *Boiss., *F. insularis *H. Wolff
46	*F. thyrsifolia *(Sm.) Koch	*Ferula thyrsifolia *Sm.
47	*F. trojana *Akalın & Pi	
48	*F.* *vesceritensis* Coss. & Durieu ex Batt.	

**Table:** 

**Plant name **	**Yield (%)**	**Methods**	**Part**	**Main components (%)**	**Class**	**Ref.**
*F. macedonica*	-	HD	Inflorescence	α-Pinene (43.1), Sabinene (26.7)	Monoterpene (69.8)	(29)
	-		Aerial parts	α-Pinene (22.8), Sabinene (15.5)	Monoterpene (38.3)	
*F. angulata*	2.8	HD	Aerial parts	α-Pinene (15.4), cis-Ocimene (30)	Monoterpene (45.5)	(30)
	0.66		Flowers	β-Phellandrene (16.5), α-Phellandrene (27), p-Cymene (10), α-Pinene (12)	Monoterpene (65.5)	(31)
	0.54		Stems	β-Phellandrene (16), α-Phellandrene (18), p-Cymene (17.7), α-Pinene (21)	Monoterpene (72.7)	
	0.43		Leaves	α-Pinene (16.8), α-Phellandrene (20.7), p-Cymene (14.5), β-Phellandrene (16)	Monoterpene (68)	
	2.65	HD	Fruits	Limonene (38), α-Pinene (18.1)	Monoterpene (56.1)	(32)
	6.5	MAHD		Limonene (35), α-Pinene (14)	Monoterpene (49)	
	-			β-Phellandrene (32), α-Phellandrene (13.8)	Monoterpene (45.8)	(33)
*F.carduchorum*	0.63	HD	Leaves	α-Pinene (26), cis-Ocimene (24.5), Bornyl acetate (6.2)	Monoterpene (50.5), Oxygenated monoterpene (6.2)	(18)
	3.2		Fruits	cis-Ocimene (64-76), α-Pinene (7.3-15)	Monoterpene (71.3-91)	
	1.3	HD	Aerial parts	(z)-β-Ocimene (43.5), α-Pinene (18.2)	Monoterpene (61.7)	(34)
*F* *.* * phialocarpa*	0.14	HD	Aerial parts	α-Pinene (41), α-Phellandrene (14.2), β-Phellandrene (9.5)	Monoterpene (64.7)	(35)
	0.89		Inflorescence	α-Pinene (43.38), *cis*-Chrysanthenyl acetate (6) 2,4,5-Trimethyl benzaldehyde (17).	Monoterpene (43.38), Oxygenated monoterpene (6) Oxygenated hydrocarbon (17)	(36)
*F.contracta*	0.68	HD	Aerial parts	β-Phellandrene (15), (E)-β-Ocimene(10) α-Phellandrene (14.5), β-eudesmol (11)	Monoterpene (39.5), Oxygenated sesquiterpene (11)	(37)
	1.3		Flowers	β-Phellandrene (25), α-Phellandrene (25)	Monoterpene (50)	
	0.54		Leaves	β-Eudesmol (24.5 ), Spathulenol (16), Citronellol (12)	Oxygenated sesquiterpene (40.5), oxygenated monoterpene (12)	
	0.4		Stems	β-Phellandrene (15.5), α-Phellandrene (11.5)	Monoterpene (27)	
*F. macrocarpa*	-	HD	Flowers	Bornyl acetate (37.1), Terpinolene (10)	Oxygenated monoterpene (37.1), Monoterpene (10)	(38)
	-		Leaves	Bornyl acetate (37.91), o-Cymene (7.83)	Oxygenated monoterpene (37.91), Monoterpene (7.83)	
	-		Aerial parts	Bornyl acetate (45.7), Borneol (17.2)	Oxygenated monoterpene (62.9)	(39)
	0.03-0.04	HD, MAHD	Flower	Borneol and Bornyl acetate	Oxygenated monoterpene	(40)
	0.80	HD	Fruit	Bornyl acetate (49), 2,3,6-Trimethyl benzaldehyde (7)	Oxygenated monoterpene (49), Oxygenated hydrocarbon (7)	(41)
*F. blancheana*	-	HD	Flowers	Sabinene (23.2), Myrcene (17.5)	Monoterpene (40.7)	(42)
	-		Aerial parts	Bornyl acetate (11.7), β-Caryophyllene (10.2)	Oxygenated monoterpene (11.7), Sesquiterpene (10)	
	-		Roots	E)-2-Decenal (20.3), Caryophyllene oxide (17.8)	Oxygenated hydrocarbon (20.3), Oxygenated sesquiterpene (17.8)	
*F. bernardii*	0.2	HD	Aerial parts	2,4,5-Trimethylbenzaldehyde (21), α-Pinene (17)	Oxygenated hydrocarbon (21), Monoterpene (17)	(10)
	-	HD	Aerial parts	α-Pinene (35), Bornyl acetate(11.6), z-β-Ocimene (14.2)	Monoterpene (49.2), Oxygenated monoterpene (11.6)	(16)
*F. pachyloba*	1.5	HD	Aerial parts	(Z)-β-Ocimene (25.7), α-Pinene (9.8)	Monoterpene (35.5)	(43)
*F. longistylis*	0.16	HD	Aerial parts	2,3,6-trimethylbenzaldehyde (32.7), Bornyl acetate (12.6)	Oxygenated hydrocarbon (32.7), Oxygenated monoterpene (12.6)	(43)
	6.4		Fruits	2,3,6-Trimethylbenzaldehyde (29),(Z)-β-Ocimene (16), α-Pinene (17)	Oxygenated hydrocarbon (29), Monoterpene (33)	(7)
*F. isaurica*	12	HD	Fruits	α-Pinene (31.5), Limonene (24.2), Myrcene (17.0)	Monoterpene (72.7)	(44)
	0.7		Roots	Terpinolene (42.1), Myrcene (27)	Monoterpene (69.1)	
	0.08		Aerial parts	Nonacosane (25.5), Hexadecanoic acid (14.8)	Hydrocarbon (25.5), Fatty acid (14.8)	(43)
*F. syriaca*	4.8	HD	Fruits	Myrcene (15.3), terpinolene (12.5), 4,6-Guaiadiene (10.7)	Monoterpene (27.8), Sesquiterpene (10.7)	(44)
	1.1		Roots	Bornyl acetate (69.4), Terpinolene (12.5)	Oxygenated monoterpene (69.4), Monoterpene (12.5)	
*F. platycarpa*	0.07	HD	Aerial parts	2,3,6-Trimethylbenzaldehyde (29.8), cis-Chrysanthenyl acetate (24.2)	Oxygenated hydrocarbon (29.8), Oxygenated monoterpene (24.2)	(43)
*F. thyrsiflora*	0.80	HD	Aerial parts	Spathulenol (31)	Oxygenated sesquiterpene (31)	(45)
*F. sylvatica*	0.10	HD	Aerial parts	Sparhulenol (13)	Oxygenated sesquiterpene (13)	(45)
	-		Roots	2,3,6-Trimethylbenzaldehyde (92.7)	Oxygenated hydrocarbon (92.7)	(46)
	-		Aerial parts	Germacrene D (32.5)	Sesquiterpene (32.5)	
	-		Inflorescence	Myrcene (29.2)	Monoterpene (29.2)	
	-	M.D	Fruits	p-Cymene (45.8), 2,5 dimethoxy p-Cymene (40)	Monoterpene (85.8)	(47)
*F. nodosa*	3	HD	Aerial parts	α-Pinene (31)	Monoterpene (31)	(45)
	-	SDE		2,3,4-Trimethylbenzaldehyde (42.2), α-Pinene(22.5)	Oxygenated hydrocarbon (42.2), Monoterpene (22.5)	(48)
	-	SFE		α-Pinene (55.5), Myrcene (10), cis-β-Ocimene (7)	Monoterpene (72.5)	
*F. pauciradiata*	-		Fruits	trans-Chrysanthenyl acetate (25), 2,3,6-Trimethyl benzaldehyde (20.7), α-Pinene (23.7)	Oxygenated monoterpene (25), Oxygenated hydrocarbon (20.7) Monoterpene (23.7)	(20)
	-	HD	Fruits	Bornyl acetate (30.5), α-Pinene (7), Germacrene D (8)	Oxygenated monoterpene (30.5), Monoterpene (7), Sesquiterpene (8)	(49)
	-		Roots	2,5, dimethoxy-*p*-Cymene (70), *p*-Cymene (12.5)	Monoterpene (82.5)	
	-		Aerial parts	2,5, dimethoxy-*p*-Cymene (33), Nonacosane (9), α-Pinene (9)	Monoterpene (42), Hydrocarbon (9)	
*F.asparagifolia*	-	M.D	Fruits	2,3, 6-trimethylbenzaldehyde (42), α-Pinene (11)	Oxygenated hydrocarbon (42), Monoterpene (11)	(47)
	**-**	HD	Fruits	2, 3, 6-trimethylbenzaldehyde (38.9), Myrcene (18.2)	Oxygenated hydrocarbon (38.9), Monoterpene (18.2)	(50)
*F. aucheri*	-	MD	Fruits	α-Pinene (36)	Monoterpene (36)	(47)
*F. confuse*	-	MD	Fruits	p-Cymene (24), 2,5-dimethoxy-p-Cymene (63.5)	Monoterpene (87.5)	(47)
*F. galbanifera*	-	MD	Fruits	trans-Chrysanthenyl acetate (17.2), Limonene (10), p-Cymene (12), α-Phellandrene (11),	Oxygenated monoterpene (17.2), Monoterpene (33)	(47)
	1.3	HD	Fruits	α-Pinene (31.8), Sabinene (15.8), Limonene (7)	Monoterpene (54.6)	(51)
*F. humilis*		MD	Fruits	Limonene (31), (Z)-b-Ocimene (32)	Monoterpene (63)	(47)
	3.9	HD	Fruits	Limonene (17.5), (Z)-β-Ocimene (32.5), α-Pinene (12)	Monoterpene (62)	(51)
*F. campestris*	0.11	HD	Aerial parts	2,4,5-trimethyl benzaldehyde, 2,4,6-trimethyl benzaldehyde	Oxygenated hydrocarbon	(52)
	0.13		Flowers			
	0.05		Roots			
	1.50		Fruits			
	0.11	MAHD	Aerial parts			
	0.13		Flowers			
	0.10		Roots			
	1.05		Fruits			
		HD	Roots	α-Pinene (58.3–75)	Monoterpene (58.3–75)	(23)
	6.4		Fruits	Myrcene (36), α-Pinene (23), *γ*-Terpinene (10)	Monoterpene (69)	
	-	WD	Fruits	Myrcene (33.4–39.7), α-Pinene (23)	Monoterpene (56.4-62.7)	(53)
*F. idaea*	-	MD	Fruits	p-Cymene (18.5), α-Pinene (16), Carvacrol methyl ether (13), 2,3,6-Trimethyl benzaldehyde (14)	Monoterpene (34.5), Oxygenated monoterpene (13), Oxygenated hydrocarbon (14)	(47)
*F.macrosciadia*	-	MD	Fruits	p-Cymene (19.5), Carvacrol methyl ether (78)	Oxygenated monoterpene (78), Monoterpene (19.5)	(47)
*F. mughlae*	-	MD	Fruits	α-Pinene (25.4), Cubenol (12.7)	Monoterpene (25.4), Oxygenated sesquiterpene (12.7)	(47)
	-	HD	Aerial parts	α-Pinene (45.5), Camphene (10.5)	Monoterpene (56)	(54)
	-		Roots	α-Pinene (37.5), Borneol (9.5)	Monoterpene (37.5), Oxygenated monoterpene (9.5)	
	-		Fruits	α-Pinene (53), Myrcene (3.9), β-Phellandrene (11), Limonene (6)	Monoterpene (72.9)	
*F. sandrasica*	-	MD	Fruits	α-Pinene (40.8), Germacrene D (8)	Monoterpene (40.8), Sesquiterpene (8)	(47)
	-	HD	Herb	α-Pinene (26.5), Camphene (5), Caryophyllene oxide (6)	Monoterpene (31.5), Oxygenated sesquiterpene (6)	(55)
	-		Roots	α-Pinene (28), δ-3-Carene (14.2), Limonene (26)	Monoterpene (68.2)	
	0.62		Leaves	Ocimene (30.5), α-Pinene (17.8), δ-3-Carene (27.4)	Monoterpene (75.7)	(56)
	0.02	HD	Aerial parts	Limonene (29), Terpinolene (14), α-Pinene (15.6)	Monoterpene (58.6)	(57)
*F. silaifolia*	-	MD	Fruits	α-Pinene (5.5), Trans-Chrysanthenyl acetate (83.5)	Oxygenated monoterpene (83.5), Monoterpene (5.5)	(47)
*F. trachycarpa*	-	MD	Fruits	γ-Terpinene (27.8)	Monoterpene (27.8)	(47)
	-	HD	Fruits	(Z)-β- Ocimene (34.1), α-Pinene (8)	Monoterpene (42.1)	(51)
	7.3		Fruits	(Z)-p-Ocimene (30.7), Myrcene (27.7)	Monoterpene (58.4)	(58)
*F. thirkeana*	4.1	HD	Fruits	Ferulagone (64), Germacrene D (14), α-Pinene (10)	Oxygenated monoterpene (64), Sesquiterpene (14), Monoterpene (10)	(59)
	-	MD	Fruits	Ferulagone (56), Germacrene D (12), α-Pinene (9)	Oxygenated monoterpene (56), Sesquiterpene (12), Monoterpene (9)	
*F. setifolia*	0.26	HD	Aerial parts	2,4,5-Trimethyl benzaldehyde (77.8), 2,3,4-Trimethyl benzaldehyde (6.2)	Oxygenated hydrocarbon (84)	(28)
*F. subvelutina*	-	HD	Aerial parts	Limonene (27), α-Phellandrene (23.1), α-Pinene (13.3)	Monoterpene (63.4)	(60)
	-		Aerial parts	Limonene (30), Terpinolene (14), α-Pinene (15.5),	Monoterpene (58.5)	(61)
*F. stellata*	0.60	HD	Aerial parts	2,4,5-Trimethyl benzaldehyde (61.1)	Oxygenated hydrocarbon (61.1)	(60)
*F.capillaris*	-	HD	Aerial parts	α-Pinene (35.8), Limonene (30.9)	Monoterpene (66.7)	(62)
*F. trifida*	1.5	HD	Aerial parts	Isobornyl acetate (25.73), Verbenol (9.66), E-β-Caryophyllene (8.68)	Oxygenated monoterpene (35.39), Sesquiterpene (8.68)	(63)
	1.4		Flowers	(E)-β-Ocimene (37.3), α-Pinene (16.5), Bornyl acetate (9.5)	Monoterpene (53.8), Oxygenated monoterpene (9.5)	(64)
	0.8		Roots	Suberosin (20.7), Cuparene (6), β-Barbatene (6.5)	Coumarin (20.7), Sesquiterpene (12.5)	
	1.3		Stems	(E)-β-Ocimene (20.7), α-Pinene (22.6), Bornyl acetate (8.5)	Monoterpene (43.3), oxygenated monoterpene (8.5)	
	1.6		Leaves	(E)-β-Ocimene (25.7), Bornyl acetate (16.7), α-Pinene (19.6)	Monoterpene (45.3), Oxygenated monoterpene (16.7)	
	1.4		Fruits	(E)-β-Ocimene (30.5), Bornyl acetate (11), α-Pinene (18)	Monoterpene (48.5), Oxygenated monoterpene (11)	

HD: Hydrodistillation; SDE: Simultaneous Distillation Extraction, MAHD: Microwave-assisted distillation.

**Table 3:** Chemical compounds isolated from the *Ferulago* genus

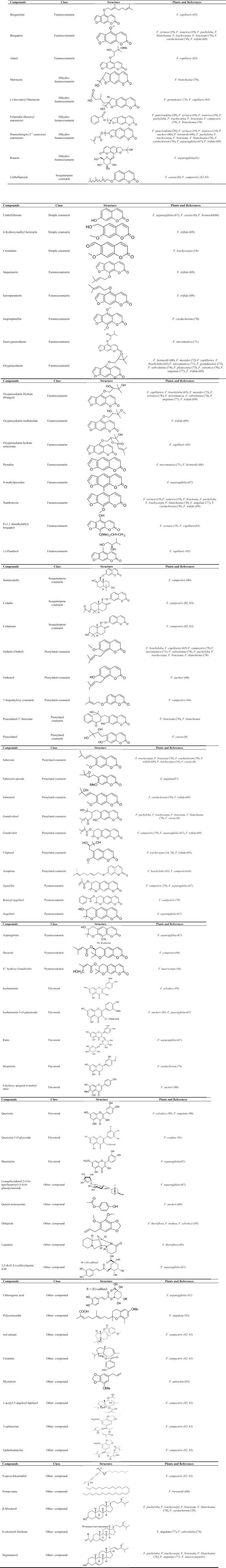

**Table 4:** Pharmacological activities and model of study of the genus *Ferulago*

**Pharmacological activities**	**Plant**	**Model**	**References**
Anti-microbial	*F. sandrasica*	*In-vitro*	(55)
	*F. pachyloba, F. blancheana, F. trachycarpa, F. bracteata*	*In-vitro*	(78)
Anti-fungal	*F. capillaris*	*In-vitro*	(62)
Anti-oxidant	*F. carduchorum*	*In-vitro*	(97)
	*F. bernardii*	*In-vitro*	(16)
	*F. trifida, F. sandrasica, F.macedonica,*	*In-vitro*	(64)
	*F. cassia*	*In-vitro*	(8)
	*F. angulata, and F. campestris*	*In-vitro*	(79)
	*F. subvelutina*	*In-vitro*	(74)
Alzheimer	*F. subvelutina, F. angulata*	*In-vitro*	(98)
	*F.pauciradiata*	*In-vitro*	(20)
	*F. carduchorum*	*In-vitro*	(34)
	*F. isaurica and F. syriaca.*	*In-vitro*	(19)
	*F. trifida*	*In-vitro*	(64)
	*F. angulata*	*In-vitro*	(99)
	*F. pauciradiata*	*In-vitro*	(20)
Anti-diabetic	*F. blancheana, F. pachyloba*, *F. trachycarpa*	*In-vitro*	(100)
Anti-coagulant	*F. carduchorum*	*In-vivo*	(17)
Anti-malaria	*F. carduchorum*	*In-vitro*	(101)
	*F. trifida*	*In-vitro*	(102)
Aphrodisiac	*F. syriaca*	*In-vitro*	(103)

## Conclusion and future perspective

To date, around 73 molecules, including coumarin, furanocoumarin, flavonoids, and stilbene have been isolated from the *Ferulago* species. Among the isolated constituents, mostly are phenolic compounds in which three simple coumarins, sixteen furanocoumarins, five dihydro-furanocoumarin, four sesquiterpene coumarin, twelve prenylated coumarins, six pyranocoumarin, nine flavonoids, and eighteen miscellaneous compounds. Coumarins and their derivatives are considered to be important taxonomic markers of the genus *Ferulago*. In the pharmacological effect part, we have discussed various studies to evaluate the ethnomedicinal uses and this has shown that the isolated constituents and different extracts from the *Ferulago* species possess several pharmacological effects including antibacterial, antioxidant, Alzheimer, anti-diabetics, anti-malaria, anti-coagulant, aphrodisiac effects. Accessible information regarding *Ferulago* spp. allows us to explore their potential benefits, highlight the gaps in our knowledge, and conduct future researches to develop new drugs. It can be concluded that the pharmacological study of *Ferulago* spp. is in agreement with the ethnomedicinal uses of the plants. 
